# Frequency of Spondyloarthropathy in Patients with Celiac Disease

**DOI:** 10.5152/ArchRheumatol.2025.25041

**Published:** 2026-04-10

**Authors:** Emrah Koç, Fatih Albayrak, Ünal Taşdemir, Hande Ece Öz, Nimet Yılmaz, Muhammed Sait Dağ, Bünyamin Kısacık

**Affiliations:** 1 Department of Rheumatology, Adana City Training and Research Hospital, Adana, Türkiye; 2 Department of Rheumatology, Dr. Ersin Arslan Training and Research Hospital, Gaziantep, Türkiye; 3 Department of Gastroenterology, Dr. Ersin Arslan Training and Research Hospital, Gaziantep, Türkiye; 4 Department of Gastroenterology, Sanko University Faculty of Medicine, Gaziantep, Türkiye; 5 Department of Rheumatology, Sanko University Faculty of Medicine, Gaziantep, Türkiye

**Keywords:** Celiac disease, inflammatory back pain, sacroiliitis, spondyloarthropathies

## Abstract

**Background/Aims:**

Celiac disease (CD) is a chronic autoimmune systemic disease characterized by extraintestinal involvement. The existing pathogenic mechanisms of CD may lead to the development of spondyloarthropathies. The aim was to determine the frequency of spondyloarthropathy in CD.

**Materials and Methods:**

Celiac patients followed up in the gastroenterology clinic were evaluated clinically and radiologically by rheumatologists. Patients with spondyloarthropathy were recorded.

**Results:**

: A total of 82 patients (20 male, 62 female) were included. The mean duration of CD was 4.85 (SD ± 6.28) years. Inflammatory low back pain was found in 43.9% of patients. Of the 36 patients who underwent sacroiliac magnetic resonance imaging (MRI), 12 (33.3%) had sacroiliitis. Among these, 4 patients (33.3% of those with sacroiliitis, 4.9% of the total cohort) had active sacroiliitis, and 8 (66.7% of those with sacroiliitis, 9.8% of the total cohort) had chronic sacroiliitis. Human Leukocyte Antigen B27 (HLA-B27) positivity was not significantly associated with MRI findings or clinical activity in the cohort. Two patients had knee monoarthritis.

**Conclusions:**

The frequency of spondyloarthropathy may be associated with factors such as persistent antigenic stimulation of the intestinal mucosa, disruption of the mucosal barrier, T lymphocyte activation, and intestinal dysbiosis in CD. A notable frequency was observed in inflammatory low back pain and sacroiliitis in patients with CD was found.

**Main Points**

- Inflammatory back pain was observed in 43.9% of celiac patients.
- Magnetic resonance imaging–confirmed sacroiliitis was detected in 14.6% of the total celiac cohort and 33.3% of those with inflammatory back pain.
- Active sacroiliitis was present in 4.9% of the total cohort.
- The association appears to be independent of HLA-B27 status.
- Findings support the importance of the gut-joint axis in the pathogenesis of spondyloarthropathy in celiac disease.

## Introduction

Celiac disease (CD) is a chronic, autoimmune-mediated enteropathy triggered by dietary exposure to gluten in genetically predisposed individuals. In celiac patients, gluten ingestion leads to the activation of both innate and adaptive immunity, resulting in chronic inflammation characterized by changes in mucosal structure, including lymphocyte infiltration, villous atrophy, and crypt hyperplasia. These structural changes in intestinal tissue lead to a loss of mucosal function and subsequent food malabsorption.^1^ Although the global prevalence is reported to be between 0.5% and 1%, it is estimated to be higher considering the presence of asymptomatic patients.^2^

Clinical findings may include gastrointestinal symptoms such as diarrhea, constipation, and abdominal pain, as well as extraintestinal manifestations like myalgia, arthralgia, and arthritis. There are also a limited number of reports on inflammatory low back pain and sacroiliitis.^3^ The association between CD and spondyloarthropathies (SpA) is thought to be rooted in a shared pathogenic basis, often referred to as the “gut-joint axis.” This concept highlights the complex interplay between intestinal health and joint inflammation. Key mechanisms include increased intestinal permeability, which allows microbial products to enter the bloodstream and trigger systemic inflammation, and intestinal dysbiosis (an imbalance in the gut microbiome), which can promote pro-inflammatory immune responses.^4^^,^^5^ Furthermore, genetic predispositions and molecular mimicry, where the immune system mistakenly attacks joint tissues due to their resemblance to gut-related antigens, are also considered contributing factors.^6^

While the link between gut inflammation and peripheral arthritis is well-documented, the connection to axial spondyloarthropathy in CD is less explored. Given these potential pathogenic links, the study aimed to determine the frequency of spondyloarthropathy and its clinical correlations in patients with CD.

## Material and Method

### Study Design and Patient Population

This cross-sectional, prospective study included 82 patients with a confirmed diagnosis of CD, who were consecutively enrolled from the gastroenterology outpatient clinic between 2021 and 2022. The diagnosis of CD was established based on positive serology (anti-tissue transglutaminase and/or anti-endomysial antibodies) and/or duodenal biopsy findings consistent with the Marsh classification.

Inclusion criteria were a confirmed diagnosis of CD and age 18 years or older.

Exclusion criteria were a previous diagnosis of another systemic inflammatory disease that could cause similar musculoskeletal symptoms (e.g., rheumatoid arthritis, systemic lupus erythematosus), a history of malignancy, and refusal to provide informed consent. Patients with gluten intolerance but negative biopsy or autoantibody tests were also excluded. Other potential causes of low back pain, such as degenerative disc disease or mechanical back pain, were ruled out based on clinical evaluation and, when necessary, imaging.

### Clinical and Rheumatological Assessment

All patients were evaluated by a rheumatologist for musculoskeletal symptoms, including inflammatory back pain, peripheral arthritis, and enthesitis. Inflammatory back pain was defined according to the Calin criteria^7^ and the Assessment of Spondyloarthropathy International Society expert criteria.^8^ A standardized questionnaire was used to collect data on demographic characteristics, duration of CD, and musculoskeletal symptoms. Clinical scores were assessed by Visual Analog Scale and Health Assessment Questionnaire.

### Radiological Evaluation

Patients presenting with inflammatory back pain underwent a sacroiliac magnetic resonance imaging (MRI) scan to assess for sacroiliitis. The MRI scans were interpreted by a single radiologist who was blinded to the patients’ clinical information.

Active sacroiliitis was defined by the presence of bone marrow edema on Short Tau Inversion Recovery sequences.

Chronic sacroiliitis was defined by the presence of structural changes such as sclerosis, erosions, or fat deposition in the absence of bone marrow edema.

### Laboratory Tests

Laboratory data, including erythrocyte sedimentation rate and C-reactive protein (CRP) levels, were recorded. The reference value for CRP was <5 mg/L. HLA-B27 status was determined for all patients with inflammatory back pain. Rheumatoid factor and anti-cyclic citrullinated peptide (anti-CCP) antibodies were measured to exclude rheumatoid arthritis; levels >20 IU/mL and >15.6 U/mL, respectively, were considered positive and were part of the exclusion criteria for this study.

### Definition of Celiac Disease Activity

Active CD was defined in newly diagnosed patients presenting with active gastrointestinal symptoms (abdominal pain, diarrhea, bloating) and/or weight loss.

Celiac disease in remission was defined in patients who were on a strict gluten-free diet for at least 1 year and were asymptomatic.

### Statistical Analysis

Statistical analysis was conducted using IBM SPSS Statistics for Windows, version 20.0 (IBM SPSS Corp.; Armonk, NY, USA). Descriptive statistics were presented as mean ± standard deviation (SD) for continuous variables and as frequency and percentage for categorical variables. The Shapiro–Wilk test was used to assess the normality of distribution for continuous variables. For group comparisons, the Mann–Whitney *U*-test was used for non-parametric continuous variables, and the chi-square test or Fisher’s exact test was used for categorical variables. Subgroup analyses were performed to compare the findings between patients with active vs. inactive CD and between HLA-B27-positive vs. -negative patients. A *P*-value less than .05 was considered statistically significant.

### Ethical Considerations

This study was approved by the Gaziantep City Hospital Non-Interventional Clinical Research Ethics Committee (Decision No: 19/2024, Date: May 15, 2024). Written informed consent was obtained from all participants prior to their inclusion in the study.

## Results

A total of 82 patients with CD were included in the study, with a female-to-male ratio of 3.1 : 1 (62 females, 75.6%; 20 males, 24.4%). The mean age was 38.4 ± 12.1 years. The median duration of CD was 4 years (interquartile range: 1-8 years), with a mean of 4.85 ± 6.28 years, indicating a wide distribution. Demographic and clinical characteristics are summarized in Table 1.

Of the 82 patients, 25 (30.5%) had active CD at the time of evaluation, while 57 (69.5%) were in remission on a gluten-free diet. Inflammatory back pain was present in 36 patients (43.9%). Other musculoskeletal findings included arthralgia in 15 patients (18.3%) and peripheral monoarthritis of the knee in 2 patients (2.4%).

### Radiological and Laboratory Findings

All 36 patients with inflammatory back pain underwent sacroiliac MRI. Sacroiliitis was detected in 12 of these patients (33.3%), corresponding to a prevalence of 14.6% in the total cohort of 82 celiac patients.

Active sacroiliitis was found in 4 of the 12 patients with MRI findings (33.3%), representing 4.9% of the total cohort.

Chronic sacroiliitis was observed in the remaining 8 patients (66.7%), representing 9.8% of the total cohort.

HLA-B27 was positive in 3 of the 36 patients (8.3%) who were tested. There was no statistically significant association between HLA-B27 positivity and the presence of active or chronic sacroiliitis (*P* > .05).

### Subgroup Analysis

Clinical and radiological findings were compared between patients with active and inactive CD. Patients with active CD had a significantly higher frequency of inflammatory back pain compared to those in remission (60.0% vs. 36.8%, *P* = .041). However, there was no significant difference in the prevalence of MRI-confirmed sacroiliitis between the 2 groups (*P* > .05). The mean duration of CD was shorter in patients who described inflammatory low back pain compared to those without (*P* = .013).

Among other rheumatologic conditions, 1 patient had a pre-existing diagnosis of rheumatoid arthritis, 1 had psoriatic arthritis, 1 had familial Mediterranean fever, and 1 had psoriasis. Four patients had been diagnosed with ankylosing spondylitis prior to their CD diagnosis.

## Discussion

In this study, the frequency of spondyloarthropathy was investigated in a cohort of 82 CD patients. The findings reveal a notable prevalence of rheumatologic symptoms, with inflammatory back pain affecting 43.9% of patients and MRI-confirmed sacroiliitis present in 14.6% of the total cohort. These results highlight a significant and potentially under-recognized overlap between CD and axial inflammation.

The prevalence of sacroiliitis in the study is higher than that reported in the general population, suggesting a true association. The findings are consistent with several previous studies. For instance, Vereckei et al reported sacroiliac involvement in 24% of CD patients with low back pain using bone scintigraphy.^9^ However, the prevalence is lower than the 63% reported by Usai et al in a smaller study that also utilized scintigraphy.^10^ These differences may be attributable to variations in imaging modality (MRI vs. scintigraphy), patient selection, and genetic backgrounds. The use of MRI, the gold standard for detecting sacroiliitis, provides a more specific assessment of both active and chronic inflammation.

The pathogenic link between CD and spondyloarthropathy is best explained by the “gut-joint axis” theory.^4^^,^^5^ Chronic intestinal inflammation in CD leads to increased intestinal permeability, allowing luminal antigens and microbial products to translocate into the systemic circulation. This process can trigger a systemic inflammatory response, leading to extraintestinal manifestations, including joint inflammation. The activation of T cells and pro-inflammatory cytokines like Tumor Necrosis Factor alpha (TNF-α) and Interleukin-17 (IL-17) in the gut mucosa, which are also pivotal in the pathogenesis of SpA, likely plays a central role.^11^ The observation that patients with active CD had a higher frequency of inflammatory back pain supports this hypothesis, suggesting that ongoing gut inflammation may be a key driver of axial symptoms.

Interestingly, a significant association between HLA-B27 positivity and sacroiliitis was not found. While HLA-B27 is a strong risk factor for ankylosing spondylitis in the general population, its role in CD-associated spondyloarthropathy appears to be less pronounced. This suggests that other genetic or environmental factors, possibly related to the underlying autoimmune process of CD itself, may be more important in this specific patient population. This finding aligns with the growing understanding that SpA is a heterogeneous condition with multiple potential triggers.^6^

### Clinical Implications

The findings have important clinical implications. They suggest that a significant subset of CD patients may have or develop spondyloarthropathy. Therefore, gastroenterologists should be vigilant for musculoskeletal symptoms, particularly inflammatory back pain, in their CD patients. Conversely, rheumatologists should consider screening for CD in patients with spondyloarthropathy, especially when gastrointestinal symptoms are present or when the clinical picture is atypical. A low threshold for performing sacroiliac MRI in CD patients with persistent inflammatory back pain seems warranted, as it can lead to an earlier diagnosis and treatment of axial disease, potentially preventing long-term structural damage.

### Limitations

The primary limitation of the study is the absence of a matched control group from the general population. This prevents us from calculating a precise relative risk and definitively concluding that the frequency of spondyloarthropathy is increased. Secondly, the cross-sectional design limits the ability to establish causality or track the progression of symptoms over time. A longitudinal study would be necessary to better understand the natural history of spondyloarthropathy in CD. Finally, while subgroup analyses were performed, the relatively small sample size may have limited the statistical power to detect smaller associations, for instance, regarding the impact of gluten-free diet duration on musculoskeletal symptoms.

In conclusion, the study demonstrates a high frequency of inflammatory back pain and MRI-confirmed sacroiliitis in patients with CD, supporting a significant clinical overlap between these 2 conditions. The pathogenesis is likely multifactorial, involving the gut-joint axis, but does not appear to be strongly dependent on HLA-B27 status. Increased awareness and interdisciplinary collaboration between gastroenterologists and rheumatologists are crucial for the early diagnosis and optimal management of these patients. Further large-scale, prospective studies with control groups are needed to confirm these findings and to elucidate the precise mechanisms underlying this association.

## Data Availability Statement:

The data that support the findings of this study are available on request from the corresponding author.

## Ethics Committee Approval:

Ethical committee approval was received from the Ethics Committee of Gaziantep City Hospital (Approval No.: 19/2024; Date: May 15, 2024).

## Artificial Intelligence Usage Statement:

The authors declared that ChatGPT has been used for improving language and editing grammar in the preparation of the manuscript.

## Informed Consent:

Written informed consent was obtained from the participants who agreed to take part in the study.

## Peer-review:

Externally peer-reviewed.

## Author Contributions:

Concept – B.K., E.K.; Design – E.K., F.A.; Supervision – B.K., E.K.; Resources – E.K.; Materials – E.K.; Data Collection and/or Processing – B.K., E.K., F.A., Ü.T., H.E.Ö., N.Y., M.S.D.; Analysis and/or Interpretation – B.K., E.K.; Literature Search – E.K.; Writing – E.K.; Critical Review – B.K., E.K.

## Declaration of Interests:

The authors have no conflicts of interest to declare.

## Funding:

The authors declare that this study received no financial support.

## Figures and Tables

**Table 1.** Demographic and Clinical Characteristics of Celiac Disease Patients (n = 82)

**Characteristic**	**Total Cohort (n = 82)**	**Patients with IBP (n = 36)**	**Patients without IBP (n = 46)**	*P*
Age (years), mean ± SD	38.4 ± 12.1	39.1 ± 11.5	37.8 ± 12.8	>.05
Female, n (%)	62 (75.6)	28 (77.8)	34 (73.9)	>.05
CD duration (years), median (IQR)	4 (1-8)	2.5 (1-6)	5 (2-10)	.013
Active CD, n (%)	25 (30.5)	15 (41.7)	10 (21.7)	.041
Arthralgia, n (%)	15 (18.3)	8 (22.2)	7 (15.2)	>.05
Peripheral arthritis, n (%)	2 (2.4)	1 (2.8)	1 (2.2)	>.05
HLA-B27 positive, n (%)	3 (8.3)	3 (8.3)	N/A	—
MRI sacroiliitis, n (%)	12 (33.3)	12 (33.3)	N/A	—
Active sacroiliitis	4 (11.1)	4 (11.1)	N/A	—
Chronic sacroiliitis	8 (22.2)	8 (22.2)	N/A	—

Comparative analysis of demographic and clinical characteristics between patients with and without inflammatory back pain. Percentages for HLA-B27 and MRI findings are calculated out of the subgroup of patients with IBP (n = 36).

CD, celiac disease; IBP, inflammatory back pain; IQR, interquartile range; MRI, magnetic resonance imaging.
